# Indications and determinants of caesarean section delivery: Evidence from a population-based study in Matlab, Bangladesh

**DOI:** 10.1371/journal.pone.0188074

**Published:** 2017-11-20

**Authors:** Tahmina Begum, Aminur Rahman, Herfina Nababan, Dewan Md. Emdadul Hoque, Al Fazal Khan, Taslim Ali, Iqbal Anwar

**Affiliations:** 1 Health Systems and Population Studies Division, International Centre for Diarrhoeal Disease Research Bangladesh (icddr,b), Dhaka, Bangladesh; 2 Maternal and Child Health Division, International Centre for Diarrhoeal Disease Research Bangladesh (icddr,b), Dhaka, Bangladesh; 3 Nossal Institute for Global Health, School of Population and Global Health, the University of Melbourne, Melbourne, Australia; 4 Nutrition and Clinical Services Division, International Centre for Diarrhoeal Disease Research Bangladesh (icddr,b), Dhaka, Bangladesh; University of North Carolina at Chapel Hill, UNITED STATES

## Abstract

**Background and methods:**

Caesarean section (C-section) is a major obstetric intervention for saving lives of women and their newborns from pregnancy and childbirth related complications. Un-necessary C-sections may have adverse impact upon maternal and neonatal outcomes. In Bangladesh there is paucity of data on clinical indication of C-section at population level. We conducted a retrospective study in icddr,b Health and Demographic Surveillance System (HDSS) area of Matlab to look into the indications and determinants of C-sections. All resident women in HDSS service area who gave birth in 2013 with a known birth outcome, were included in the study. Women who underwent C-section were identified from birth and pregnancy files of HDSS and their indication for C-section were collected reviewing health facility records where the procedure took place, supplemented by face-to-face interview of mothers where data were missing. Indications of C-section were presented as frequency distribution and further divided into different groups following 3 distinct classification systems. Socio-demographic predictors were explored following statistical method of binary logistic regression.

**Findings:**

During 2013, facility delivery rate was 84% and population based C-section rate was 35% of all deliveries in icddr,b service area. Of all C-sections, only 1.4% was conducted for Absolute Maternal Indications (AMIs). Major indications of C-sections included: repeat C-section (24%), foetal distress (21%), prolonged labour (16%), oligohydramnios (14%) and post-maturity (13%). More than 80% C-sections were performed in for-profit private facilities. Probability of C-section delivery increased with improved socio-economic status, higher education, lower birth order, higher age, and with more number of Antenatal Care use and presence of bad obstetric history. Eight maternal deaths occurred, of which five were delivered by C-section.

**Conclusions:**

C-section rate in this area was much higher than national average as well as global recommendations. Very few of C-sections were undertaken for AMIs. Routine monitoring of clinical indication of C-section in public and private facilities is needed to ensure rational use of the procedure.

## Background

Caesarean section (C-section) is a major obstetric intervention introduced in late Nineteenth century to save lives of women and their newborns from life-threatening pregnancy and childbirth related complications [1]. The population-based C-section rate is considered as a process indicator in maternal health to monitor progress [2]. World Health organisation (WHO) has recommended that the population based C-section rate should lie between 5 and 15 percent [3] to have an optimal impact [4, 5]. Nevertheless, the past decade has observed a tremendous increase in population based all-cause C-section rates globally. Recent data from both developed and developing countries have documented the average rate of 27% C-section during year 2013 [6, 7]. Unnecessary C-section may have adverse impact upon maternal, neonatal and infant morbidity and mortality. Moreover, high cost of C-section may result catastrophic health expenditure for families and exert additional pressure upon overburdened health systems particularly in low and middle income countries [2, 7]. Remarkably, non-medical indications constitute one-third of total 18.5 million C-sections performed annually, contributing heavily to the global total excess of C-section [3]. The alarming high C-section rate warrants monitoring indications of all C-sections in public and private facilities [8].

Bangladesh could make remarkable progress in safe motherhood where MMR has reduced from 322 deaths per 100,000 live births in 2001 to 194 deaths per 100,000 live-births in 2010. Access to basic and comprehensive emergency obstetric care (EmOC) has increased; however contributed mainly by the for-profit private sector facilities. Bangladesh Demographic and Health Survey (BDHS) 2014 shows that of 37% facility delivery, 60% took place in for-profit private sector hospitals [9]. Important to note that C-section rate has increased from 4% in 2004 to 23% in 2014 [10]. This high and rising C-section rate is certainly a cause of concern, however, provide little information on how or why C-section rate is increasing and what should be done. Both demand and supply side factors are attributed for this rapid rise in population-based C-section rate in different contexts. The ongoing demand side financing (DSF) maternal health voucher scheme might have some impact on rising C-section rate [11, 12]. However, DSF scheme covers only 53 subdistricts out of total 490 [11]. Other factors attributed for high and rising C-section rates include recent progress in social determinants of health, improvement in road-transportation system, and the extensive growth of for-profit private facilities capable to provide comprehensive EmOC services [11]. A number of global studies have explored poor quality of care maternal health. [13]. Bangladesh is not an exception in this regard; both technical and perceived quality of Maternal and Neonatal Health (MNH) care is poor in both public and private facilities[14, 15] Many of the private health facilities do not have a full range of basic emergency obstetric care (EmOC) services, lack many necessary equipment, and do not follow good medical practices [16, 17].

Though, a number of studies have explored trends and inequities in use of maternal health care services [11, 12, 18], there is paucity of data on clinical indications for caesarean in Bangladesh particularly from population based studies, essential for deeper understanding of why caesarean delivery rate is increasing and what strategies are needed to control the epidemic of C-sections. The present study aims to explore the indications of C-section along with their socio-demographic determinants in Matlab, Bangladesh, to inform policy for strategies for ending preventable maternal and neonatal mortality [19].

## Methods and materials

### Study setting

**Fig 1:**
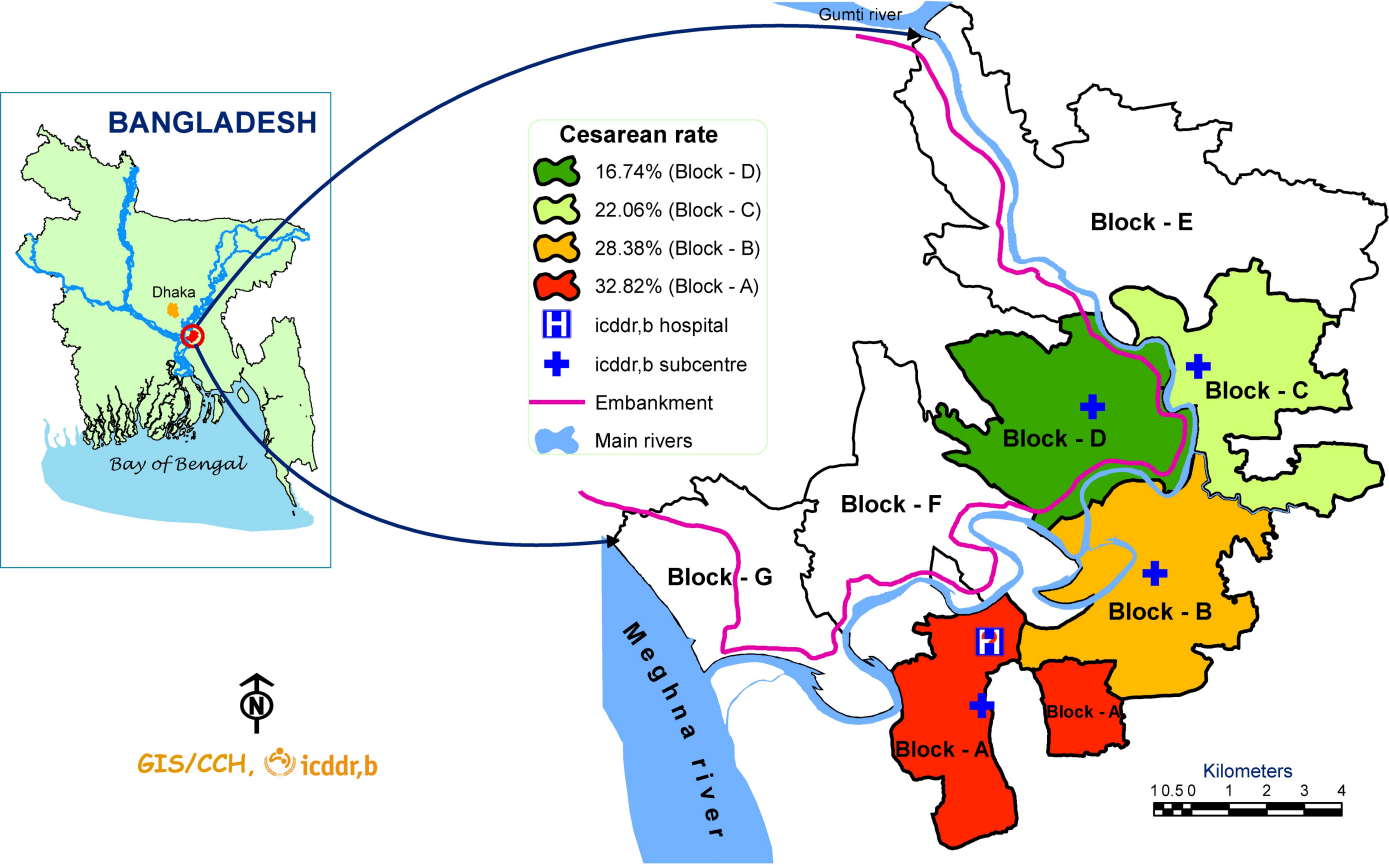
Study area in Matlab with population level C-section rate.

Matlab is a rural sub-district under Chandpur district located about 55 km Southeast of Dhaka, the capital of Bangladesh. International Centre for Diarrhoeal Disease Research, Bangladesh (icddr,b) has been maintaining a Health and Demographic Surveillance System (HDSS) in this area covering a population of 230,000 (in 2013) since 1966 [20]. Matlab HDSS area is divided into 2 equal halves: icddr,b service area (SA) and government service area (Fig 1). The present study was conducted in Matlab icddr,b service area (divided further into 4 blocks, A to D) where under a maternal, neonatal and child health (MNCH) program, free basic emergency obstetric, neonatal and child health care services are provided from four icddr,b sub-centres and one central icddr,b Matlab hospital (Fig 1) [15]. In addition to icddr,b services, basic and comprehensive emergency obstetric and neonatal care (EmONC) services are available from public sector subdistrict (Matlab) and district hospitals (Chandpur), and from a growing number of for-profit private sector hospitals and clinics situated mostly in Chandpur district town [15].

### Study design and data collection method

This is a retrospective study where all women who delivered in icddr,b service area during 2013 were included in the analysis. Several existing HDSS databases were linked to retrieve required socio-demographic, service use and the birth outcome data to perform the statistical analysis to look into the indications and socio-demographic determinants of C-section. We used the following HDSS data files: a) birth file, b) pregnancy registration file, c) the socioeconomic census 2014, and d) verbal autopsy database. Women who delivered in 2013 were identified from the HDSS birth file. Service utilization and birth-outcome data were available from pregnancy registration file, birth file and icddr,b facility records (four sub centres and icddr,b Matlab hospital).While information on household construction materials, source of drinking water, type of latrine use and other asset variables were available from the Matlab Icddr,b 2014 socio economic census, which were subsequently used to construct wealth quintiles using Principal Component and Factor Analysis Methods [21, 22]. The number and causes of maternal deaths were gathered from routine verbal autopsy forms of the HDSS. All monitoring, surveillance and socioeconomic survey databases were linked together using HDSS assigned unique registered identification number (RID) of pregnant women to perform required analysis.

All women who delivered in icddr,b service area during 2013 were identified reviewing the birth and pregnancy files. Among them, who delivered by C-section, their indications were collected reviewing health facility records where the procedure took place. One project research physician identified the public and private facilities where C-section actually took place and visited all these facilities that include Chandpur district hospital and seven private clinics situated in Matlab and Chandpur to collect information on clinical indication of caesarean sections. Facility records review included patient admission file, in-patient registrar, operation theatre registrar, and bed-head tickets. However, the indications of C-sections for 195 women could not be retrieved following this procedure, therefore ‘obstetric complications’ recorded as referral indications in icddr,b service registrars (maintained electronically) were accepted as proxy indications for C-sections [23]. Even though, data were missing for 22 women who probably bypassed icddr,b service delivery system. Indications of C-section for these 22 women were gathered through direct interview with mothers. A female study investigator with obstetric background conducted all these 22 interviews. A structured check-list was used to guide the women to recall their birth events to retrieve true clinical indication of C-section undertaken. Interview data were validated using other proxy sources such as referral notes, doctor’s prescriptions and discharge certificates (if any). All C-section indications data were entered in a data base and linked with other HDSS databases using unique HDSS identifier (RID of the mother). WHO, International Classification of Disease version 10 (ICD 10) [24] was used to classify the indication of C-sections. Field data collection was completed in 4 months during September to December 2014. One programmer assisted in data entry, cleaning and linking with other HDSS databases.

### Data analysis

Indications of C-sections were first presented as frequency distribution of individual clinical conditions (ICD-10). These indications (of C-section) were further divided into different groups following three distinct classification systems: 1) Absolute vs. Non-Absolute Maternal Indications 2) Primary vs. Repeat C-sections, and 3) Maternal vs. Fetal indications; to identify the specific risk-groups for C-section.

**Fig 2:**
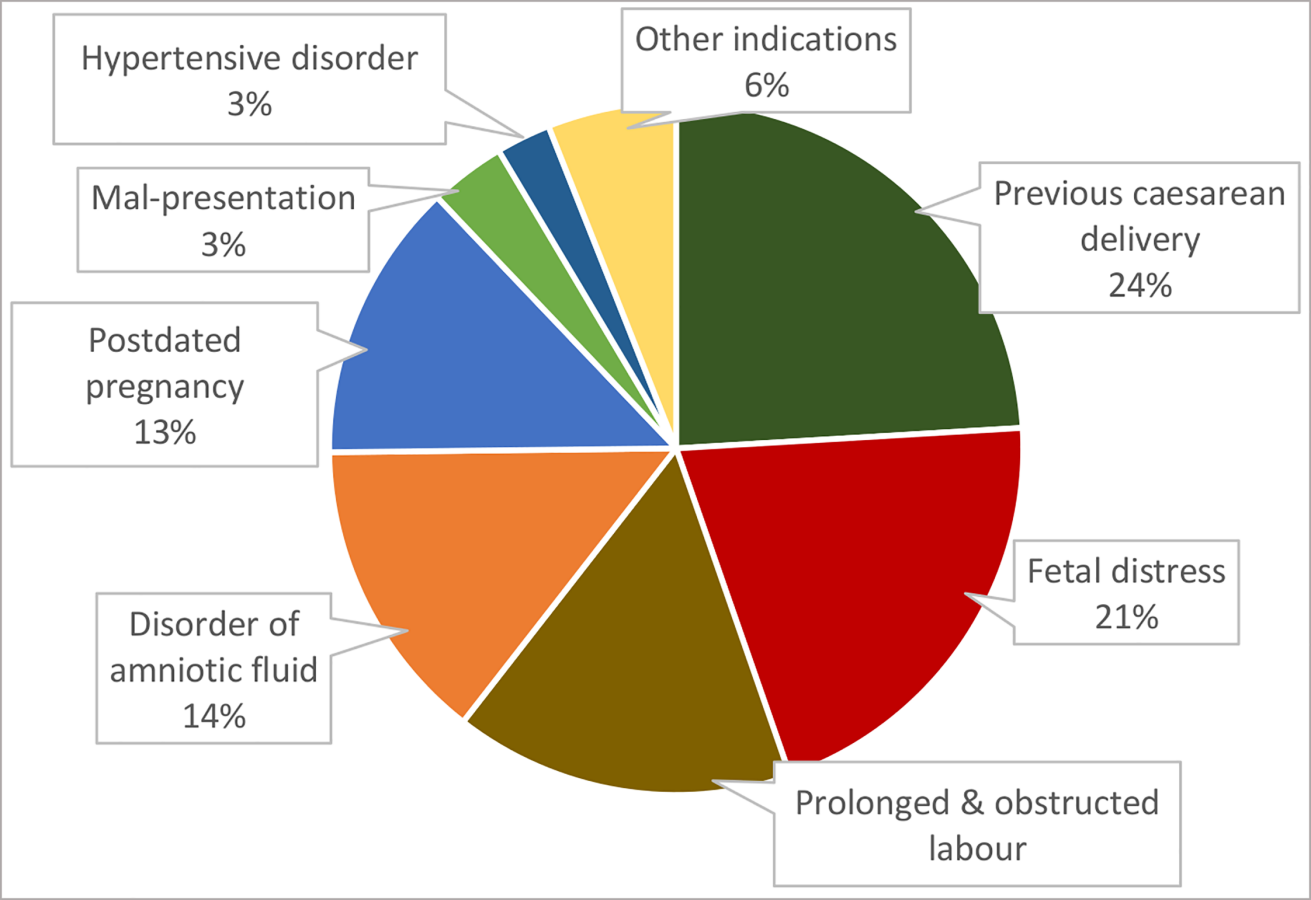
Distribution of C-section indications under eight broad categories according to ICD-10 code.

ICD-10 codes for indication of C-section were grouped into 10 subclasses: hypertensive disorder, mal presentation, disorder of amniotic fluid, antepartum haemorrhage (APH) including placenta praevia, post-dated pregnancy, prolonged & obstructed labour, fetal distress, previous caesarean delivery, maternal disorder related to pregnancy, and general disease complicating pregnancy. The fetal distress group includes non-specified fetal distress and fetal distress due to meconium-stained liquor. Hypertensive disorder covers gestational hypertension, pre-eclamptic toxaemia (PET) and eclampsia. Amniotic fluid disorder category includes oligo and poly-hydramnion. However, cephalo-pelvic disproportion (CPD) and failed induction of labor are included in the category of prolonged or obstructed labor. Finally, C-section indications documented as Maternal distress, Rh negative mothers and retention of urine were grouped under ‘pregnancy-related maternal disorder’, while thalassemia, anaemia, asthma belong to ‘general disease complicating pregnancy’ category. Later on, C-section indication documented under APH including placenta praevia’ ‘pregnancy-related maternal disorder’ and ‘general disease complicating pregnancy’ were re-categorised under ‘other indication’ (Fig 2)

AMIs include 4 distinct life-threatening obstetric complications: uncontrolled bleeding, unstable lie or presentations (transverse lie, face or brow presentation), gross cephalo-pelvic disproportion (CPD) and uterine rupture [25].

Primary C-section group included women having C-section for the first time, while repeat C-section group included women with one or more C-section prior to current birth.

Maternal causes included clinical conditions such as hypertensive disorder, amniotic fluids disorders, post-dated pregnancy, maternal distress, Rh-negative mother, psychological disorder and general diseases complicating pregnancies such as thalassemia, anaemia, asthma, and retention of urine. Fetal causes included multiple gestation, big baby, and fetal distress. Problems related to both mother and fetus such as prolonged or obstructed labor, CPD, failed induction, placenta praevia, and malpresentation were grouped as combined cause.

Further analyses focused on exploring socio-demographic determinants of C-section delivery. The exposure variables included; maternal age, religion, education, employment status, wealth quintile, birth order, number of Ante Natal Care (ANC), history of fetal loss, delivery outcome, sex of the baby, fetal number, and gestational age while the outcome variable was ‘delivery by C-section (0 = delivery by other method; 1 = delivery by C-section). At bivariate level, Chi-square tests were performed to explore the relationship between outcome and exposure variables. Multicollinearity between exposures variables were checked using Variants Inflation Factors (VIF). The exposure variables showing significant relationship with the outcome variable at bivariate level were included in the binary logistic regression model. We used Mantel-Haenszel test to examine the independent effects of exposure variables upon C-section delivery after controlling the confounding effects of all covariates in the model. The strength of association between exposure and outcome variables were measured as adjusted odds ratios (OR) with 95% confidence intervals (CI) of odds ratios. All analysis was performed in STATA 13 (STATA Corp.)

## Ethical approval

Ethical approval for the study (protocol number PR-14028) was obtained from Institutional Review Board of icddr,b. Approval from relevant hospital authorities was obtained before accessing their documents and written informed consent was taken from all mothers interviewed.

## Result

### Characteristics of women and newborn

A total of 2549 women delivered in the icddr,b service area with a known birth-outcome during 2013 were included in the study. Of them 87% were Muslims and their mean age was 26 years. More than 3/4^th^ of them completed secondary level education and only 2% were engaged in any formal job. On average 76% of this study cohort attended at least three antenatal (ANC) visits and 19% had bad obstetric histories (fetal loss in earlier pregnancies) (Table 1). Most of the newborns were live births (98.9%), singleton (99.1%), and born full-term (89.4% (Table 2).

**Table 1:** Sociodemographic characteristics of study participants.

Variables *(n = 2549)*	Frequency n (%)
***Maternal Age(Years)***	
≤19	233(9.1)
20–24	861(33.8)
25–29	700(27.5)
30–34	533(20.9)
≥35	222(8.7)
***Religion***	
Muslim	2235(87.7)
Non-Muslim	314(12.3)
***Education***	
No education	185(7.3)
Primary	462(18.1)
Secondary	1693(66.4)
Higher secondary and above	209(8.2)
***Employment status***	
Unemployed	2503(98.3)
Employed	42(1.7)
***Wealth quintiles***	
Poorest	332(16)
Poor	354(17.1)
Average	416(20)
Rich	481(23.2)
Richest	492(23.7)
***Birth order***	
1	1049(41.3)
2	793(31.2)
≥3	696(27.4)
***Number of ANC***	
0	18(0.7)
1	41(1.6)
2	550(21.6)
3	1358(53.3)
>3	582(22.8)
***History of fetal loss***	
Yes	491(19)
***Delivery place***	
Home	413(16.20)
Public	203(7.96)
Private for non profit	1095(42.96)
Private for profit	838(32.88)

**Table 2:** Birth outcome data of the study participants.

Variables *(n = 2549)*	Caesarean section	Vaginal delivery	P-value
	n (%)	n (%)	
***Sex of the newborn***			
Male	463(51.33)	833(50.58)	0.71
Female	439(48.67)	814(49.42)	
***Fetal number***			
Singleton	890(98.67)	1,635(99.27)	0.133
Multiple	12(1.33)	12 (0.73)	
***Gestational age at birth***			
Preterm(<37weeks)	98(10.86)	171(10.38)	0.7
Term(≥37 weeks)	804(89.14)	1476 (89.62)	
***Birth outcome***			0.025
Stillbirth	4(0.44)	23(1.40)	
Live birth	898(99.56)	1,624(98.60)	

### Delivery patterns

Out of 2549 deliveries, 84% were conducted in health facilities: 43% in icddr,b facilities, 33% in for-profit private facilities and 8% in government facilities. During study period, only 16% deliveries were conducted at home (Table 1). Of all deliveries 35% were conducted by C-section and the rate was highest in block A which includes Matlab town (Fig 1). Majority of C-sections (81.7%) were conducted in for-profit private sector facilities.

### Indications of C-section

Based on ICD-10 classification, ‘previous history of C-section’ was the most common indication (24.1%) for doing C-sections. Other indications included: ‘fetal distress’ 20.6%, ‘prolonged and obstructed labor’ 15.9%, ‘amniotic fluid disorder’ 14.3%, ‘post-dated pregnancy’ 13.1%, ‘maternal disorder related to pregnancy’ 4.5%, ‘fetal mal-presentation’ 3.5%, ‘hypertensive disorder in pregnancy’ 2.5%, ‘placenta praevia’ 0.78%, and ‘general disease complicating pregnancy’ 0.7% (Fig 2).

**Fig 3:**
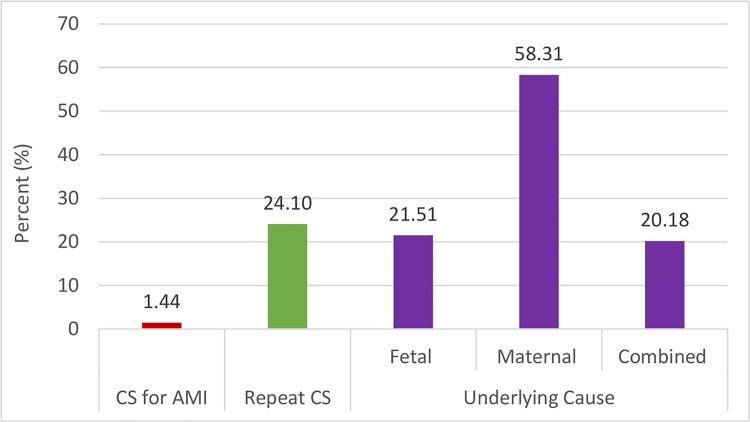
Distribution of C-section indications by three different categories. CS for AMI (Yes & No); Repeat CS (Yes & No); Underlying cause (Fetal, Maternal & combined).

Of all C-sections only 1.44% was conducted for AMIs. In another way, we found that 58.3% C-sections were conducted for maternal causes, 21.5% for fetal causes and the rest 20.2% were conducted for both maternal and fetal causes. Of 214 (24%) repeat C-section cases, there was even no secondary indication for repeating this surgical procedure. (Fig 3)

### Determinants of C-section

In multi-logistic regression model, when the effects of all co-variates were controlled statistically, probability for using C-section delivery increased with better socio-economic status, higher education of women, increasing age, decreasing birth order, and with presence of bad obstetric history. Women from richest quintile households were two and half times more likely to deliver by C-section compared to women from the poorest quintile households (OR: 2.47; 95% CI: 1.78–3.34). Similarly women with higher secondary and above education were two times (OR: 2.06 95% CI: 1.24–3.25) more likely to deliver by C-section than women with no education. Women with three or more birth order were 68% less likely to deliver by C-section (OR: 0.32 95% CI: 0.23–0.44) than women with 1^st^ birth order. Women attending more than three ANC sessions were 2 times more likely to deliver by C-section than women having ‘0–2’ ANC sessions (OR: 2.19 95% 1.67–2.82). Women having history of fetal loss were about one and half times more likely to deliver by C-section (OR: 1.38; 95% CI: 1.10–1.73) than women without bad obstetric histories. (Table 3)

**Table 3:** Determinant of caesarean delivery.

Variables	% used CS (n = 902)	Adjusted OR	P value
		(95% CI)	
**Maternal Age**			
≤19 years	34.76	1	
20–24	39.26	1.23(.91–1.73)	0.162
25–29	33.71	1.42(.97–2.13)	0.064
30–34	33.96	2.15(1.48–3.29)	0.000*
≥35 years	29.73	2.00(1.18–3.40)	0.010*
**Education Status**			
No education	21.62	1	
Primary	22.73	1.01(0.64–1.54)	0.947
Secondary	37.51	1.43(0.94–2.11)	0.084
Higher secondary and above	58.37	2.06(1.24–3.25)	0.005*
**Employment Status**			
Unemployed	35.00	1	
Employed	59.52	1.36(0.69–2.76)	0.376
**Birth order**			
1	42.55	1	
2	35.44	0.56(0.46–0.76)	0.002*
≥3	24.43	0.32(0.23–0.44)	0.000*
**Wealth Quintiles**			
Poorest	21.49	1	
Poor	23.57	0.95(0.67–1.33)	0.725
Middle	32.91	1.40(1.01–1.93)	0.061
Rich	40.76	1.90(1.37–2.58)	0.000*
Richest	48.59	2.47(1.78–3.34)	0.000*
**Number of ANC**			
0–2	26.27	1	
3	34.76	1.40 (1.11–1.76)	0.002*
>3	46.39	2.19(1.67–2.82)	0.000*
**History of fetal loss**			
No	34.69		
Yes	38.29	1.38(1.10–1.73)	0.005

* P Value < .005, Adjusting with maternal age, education status, employment status, birth order, wealth quintile, number of ANC taken & history of fetal loss

### Reported adverse maternal outcome

Verbal autopsy data reveals that during study period, a total of eight maternal deaths took place in the study area. Of them, seven took place during postpartum period and one during antepartum period due to complication of heart disease. Of seven postpartum maternal deaths, five took place after C-section and in private hospitals. None of these five deaths had AMI for undergoing C-section. The cause of post caesarean death for five mothers were as follows: two from post-partum haemorrhage, one from severe anaemia and kerosene poisoning, and the rest two from post-partum infection and pleural effusion.

## Discussion

### Main findings

In this rural area facility delivery rate was 84% and C-section rate was 35% of all deliveries. C-section rate was higher than national average (23%) [10] and even more higher than global recommendations [26]. Only 1.44% C-sections were conducted for AMIs. The top five indications were repeat C-section (24%), fetal-distress (21%), prolonged labor (16%), oligohydramnios (14%) and post-maturity (13%). In the repeat C-section group, there was no other valid indications that contribute to save lives. Higher age, lower birth order, higher education, better socioeconomic status, bad obstetric history, and three or more ANC uptake were significant predictors for C-section delivery. Of total eight maternal deaths, seven took place during postpartum period, five were among women who underwent C-section in private sector facilities.

### Interpretations

Similar to the findings in other settings [27], the population based C-section rate in the study population (35%) was higher than recommended upper limit of 15%. One recent ecological study has argued that the safe upper limit of population based C-section rate could be 19% [19]. Referring to this findings, WHO in their recent strategic document [28] has emphasized more on monitoring indications rather than concentrating on appropriate C-section rates [29, 30]. The five most common indications explored in this study have also contributed for global excess of C-section rates. One study has explored that in 2001, 30% of C-sections in developed countries were for repeat C-sections [31]. Another study in Bangladesh reported that in government hospitals 35% of C-sections were actually repeat C-sections [32]. National Institute for Health and Clinical Excellence [33] and the American College of Obstetricians and Gynaecologists [34] have clearly instructed that previous C-section should not be an indication in absence of any obstetric emergencies. There is also proof that the success rate for vaginal birth after C-section (VBAC) was much higher (80%) and morbidity rate was much lower than those with repeat C-section [29, 31]. Thus the priority should focus on reducing primary C-section rate through engaging both client and providers in informed decision making process [29, 32].

Fetal distress, identified as the second leading cause of C-section (20.6%) in this study population, has a reported global prevalence of about 20% [35] and was accounted for about 16% C-section at tertiary level hospitals in Bangladesh [32, 36]. There is a range of medical intervention, from simple maternal left lateral position, oxygen inhalation, to para-cervical amnio-infusion [37] for restoration of fetal heart rate. The reported success rate for para cervical amnio-infusion was about 70% even in limited resource setting[35] and therefore it is imperative to encourage care-givers to practice these interventions before opting for emergency C-section for fetal distress.

Prolonged labor was the next most common cause of C-section (15.9%) in the current study. It also contributed for the rising C-section rate in other population-based studies, with prevalence as high as 30% [31, 38]. Active management of second stage of labour by augmentation was found to be effective to manage prolonged labour [29, 39] without causing severe birth asphyxia[40, 41]. However, over-reporting of prolonged labour as indication of C-section could be avoided if the failed induction of labour is reported separately. In addition to that monitoring labour through plotting a simple graph such as partograph was found to reduce C-section rate by around 31%, as documented by studies done in different settings in United States, United Kingdom and South Africa [40]. In contrast to that, partograph use rate in Bangladesh is documented to be very low. A need assessment study done among 24 districts of Bangladesh has reported the partograph user rate as three percent [42]. Another study conducted in the health facilities of Khulna and Sylhet divisions of Bangladesh has conferred that partograph user rate was 5% to 33% [14].

Oligohydramnios was the indication for 14% of C-section in this study. However, a prospective cohort study done in Pakistan has documented that isolated oligohydramnios is not associated with adverse perinatal outcome such as high rate of birth asphyxia or admission to intensive care unit compare to women having normal amniotic fluid. Thus elective C-section for possible perinatal morbidity due to oligohydramnios is not recommended for any instance[43]. Moreover, it has been reported that estimated rate of oligohydramnios could be up to six percent [44] and majority of the cases the superfluous results of low amniotic fluid comes from subjective variation on sonography [45] Higher C-section for oligohydramnios in this study raises issues regarding validity of ultra-sonographic results which is common in current practice context in Bangladesh. Nevertheless, serial sonography in all four World Health Organization (WHO) recommended ANC visits are crucial to detect congenital fetal anomaly responsible for oligo hydramnions and also to identify potential threat for many fetal complication in advance. [46]. But the data from recent national demographic survey suggest that only 31% of the women in Bangladesh are receiving 4+ ANC.[9]. Furthermore, several studies have reported the poor quality of ANC mostly due to lack of physical infrastructure, more waiting time, less supportive behaviour from service providers and lack of Standard Operating Procedure (SOPs) and evidence based practice like counselling, health education [47, 48].

Similar to the finding of this study, advance maternal age has been documented as influencing factor for high C-section rates in other studies [49–53]. Certain biological changes occur with the advancement of age during pregnancy, such as mal-position, increased risk of hypertension, eclampsia, and diabetes [50]. Maternal preference together with these risks might have increased the caesarean delivery among older mother [50].

As in many other studies Socio Economic Status (SES) was found to be positively associated with C-section delivery [54]. However, the opposite trend has also been observed in developed countries, where higher education and economic status were protective against C-section as awareness and knowledge of childbirth are expected to be high among this group of women [53]. However number of ANC visit was positively associated with higher C-section delivery in this study and other studies that may raise question about its efficacy in controlling C-section epidemic [52, 55–57]. Thus the quality of ANC need to be prioritised along with the number of ANC taken with particular emphasis on engaging women on sensitive discussion on risk and benefit of both normal delivery and C-section and to provide emotional support on taking decision[58]. Though Bangladesh has already prioritised the quality ANC in their policy agenda, the inequity in service uptake has observed. The most recent study confers that 4+ ANC uptake is mostly prevalent in wealthier and educated women, and the group of women residing in urban area[59]

Majority of C-sections were conducted in for-profit private sector facilities that supports the view that private provision of care may increase C-section rate for incentive mechanism associated with the procedure [60, 61]. One study in Brazil has revealed that the prevalence of C-section delivery particularly in private sector hospitals did not correlate with obstetric risk factors [53]. In this context, clinical audit and feedback expected to have supportive role to guide the health professional in analysing and modifying their practice around evidence based clinical guideline on safe delivery care. One meta- analysis from Canadian studies has shown 13% reduction of caesarean section rate upon introduction of clinical audit and feedback and showed more positive result up to 27% C-section reduction with multifaceted intervention including second opinion and positive cultural change [62].

However, it is also evident that C-sections done regardless of obstetric risk factors, could be a means of defensive obstetric practices and that may increase the risk of maternal death particularly in low resource settings [1]. Our study supports the view as of eight maternal deaths among the study population five were related to C-section delivery with the clear evidence of poor quality of care. In relation to that WHO in their strategic document “Beyond the number” urged for verbal autopsy for all maternal deaths as every delivery has a story to tell which can provide relevant and important information for strengthening health system response [63].Bangladesh has been implementing Maternal and Perinatal Death Review (MPDR) at community and facility level through social and verbal autopsy respectively since 2010. One evaluation has shown improved quality of care and increased response to any obstetric emergency at community and facility level[64]. Currently MPDR has been implemented in the 17 out of 64 districts under the Quality improvement Initiative of government of Bangladesh [65].

### Strength and limitations

The strength of the study is that the data comes from an established and long standing health and demographic surveillance system which has enabled retrieval of entire birth cohort data for with no missing values for required analysis. All C-section indications could be captured from multiple sources. All these are strengths of the study. There was the chance for recall bias for getting indication from women where data were unavailable from facility records, given that interviews occurred one year after the birth events. However interviews were conducted by trained obstetrician with similar experiences and collected data using well-designed interview guideline and were validated with referral notes and discharge certificates available with women to overcome this weakness.

## Conclusion

Clinically non-indicated C-sections conducted in private health facilities have contributed to a high prevalence of C-section in a rural community in Bangladesh. However, over medicalization of the procedure cannot be ruled out. As repeat C-section is a dominant cause, reduction of primary C-section should be given priority. In regards to this, para-cervical amnio infusion to restore fetal distress, proper serial sonographic scanning to confirm the presence of oligo- hydramnions, monitoring labour through partograph, and if necessary augmenting labour in timely manner could be the most effective clinical interventions for reducing primary C-section rate. Dominance of private sector in provision of C-section along with increased level of adverse maternal outcome associated with C-section delivery, demand adequate policy attention to the clinical quality of MNH care in for-profit private sector facilities. Finally, a comprehensive, evidence based approach needs to be introduced to monitor indication of all C-section in public and private facilities and to motivate both provider and recipient for its rational use.
